# Marizomib for patients with newly diagnosed glioblastoma: A randomized phase 3 trial

**DOI:** 10.1093/neuonc/noae053

**Published:** 2024-03-19

**Authors:** Patrick Roth, Thierry Gorlia, Jaap C Reijneveld, Filip de Vos, Ahmed Idbaih, Jean-Sébastien Frenel, Emilie Le Rhun, Juan Manuel Sepulveda, James Perry, G Laura Masucci, Pierre Freres, Hal Hirte, Clemens Seidel, Annemiek Walenkamp, Slavka Lukacova, Paul Meijnders, Andre Blais, Francois Ducray, Vincent Verschaeve, Garth Nicholas, Carmen Balana, Daniela A Bota, Matthias Preusser, Sarah Nuyens, Fréderic Dhermain, Martin van den Bent, Chris J O’Callaghan, Maureen Vanlancker, Warren Mason, Michael Weller

**Affiliations:** Department of Neurology and Brain Tumor Center, University Hospital Zurich, Zurich, Switzerland; Department of Neurology, University of Zurich, Zurich, Switzerland; European Organisation for Research and Treatment of Cancer (EORTC), Brussels, Belgium; Department of Neurology & Brain Tumor Center, Amsterdam University Medical Centers, Amsterdam, The Netherlands; Department of Medical Oncology, University Medical Center Utrecht, University Utrecht, Utrecht, The Netherlands; Sorbonne Université, AP-HP, Institut du Cerveau – Paris Brain Institute – ICM, Inserm, CNRS, Hôpitaux Universitaires La Pitié Salpêtrière – Charles Foix, DMU Neurosciences, Service de Neurologie 2-Mazarin, Paris, France; Department of Medical Oncology, Institut de Cancerologie de L’Ouest, Saint-Herblain, France; CHU Lille, Service de neurochirurgie, Lille, France; Univ. Lille, Inserm, CHU Lille, U1192, Laboratoire Protéomique, Réponse Inflammatoire et Spectrométrie de Masse (PRISM), Lille, France; Department of Neurosurgery & Clinical Neuroscience Center, University Hospital and University of Zurich, Zurich, Switzerland; Neuro-Oncology Unit, Department of Medical Oncology, Instituto de Investigación Hospital 12 de Octubre, Madrid, Spain; Division of Neurology, Sunnybrook HSC, University of Toronto, Toronto, Ontario, Canada; Department of Radiation Oncology, Centre Hospitalier de l’Université de Montréal (CHUM), Montréal, Québec, Canada; Department of Medical Oncology, University Hospital of Liege, Liege, Belgium; Department of Oncology, McMaster University, Hamilton, Ontario, Canada; Department of Radiation Oncology, University Hospital Leipzig, Leipzig, Germany; Department of Medical Oncology, University of Groningen, University Medical Center Groningen, Groningen, The Netherlands; Department of Oncology, Aarhus University Hospital, Aarhus, Denmark; Department of Radiation Oncology, Iridium Network Antwerpen, University of Antwerp, Antwerp, Belgium; Service d’hématologie et d’oncologie, Centre intégré de cancérologie du CHU de Québec – Université Laval, Québec City, Québec, Canada; Department of Neuro-Oncology, Hospices Civils de Lyon and Université Claude Bernard Lyon 1, Lyon, France; Lyon Cancer Research Center (CRCL) UMR INSERM 1052 CNRS 5286, Lyon, France; Department of Medical Oncology, GHDC Grand Hopital de Charleroi, Charleroi, Belgium; University of Ottawa, Division of Medical Oncology, Ottawa, Ontario, Canada; Badalona Applied Research Group in Oncology (B-ARGO Group), Institut Investigació Germans Trias i Pujol (IGTP), Badalona, Spain; Chao Family Comprehensive Cancer Center and Department of Neurology, University of California, Irvine, California, USA; Division of Oncology, Department of Medicine I, Medical University of Vienna, Vienna, Austria; European Organisation for Research and Treatment of Cancer (EORTC), Brussels, Belgium; Department of Radiation Oncology, University Hospital Gustave Roussy, Villejuif, France; Brain Tumor Center at ErasmusMC Cancer Institute, Rotterdam, The Netherlands; Canadian Cancer Trials Group, Queen’s University, Kingston, Ontario, Canada; European Organisation for Research and Treatment of Cancer (EORTC), Brussels, Belgium; Princess Margaret Cancer Centre, Toronto, Ontario, Canada; Department of Medicine, Princess Margaret Cancer Centre, University of Toronto, Toronto, Ontario, Canada; Department of Neurology and Brain Tumor Center, University Hospital Zurich, Zurich, Switzerland; Department of Neurology, University of Zurich, Zurich, Switzerland

**Keywords:** EORTC 1709, glioma, MGMT, proteasome inhibitor, randomized study

## Abstract

**Background:**

Standard treatment for patients with newly diagnosed glioblastoma includes surgery, radiotherapy (RT), and temozolomide (TMZ) chemotherapy (TMZ/RT→TMZ). The proteasome has long been considered a promising therapeutic target because of its role as a central biological hub in tumor cells. Marizomib is a novel pan-proteasome inhibitor that crosses the blood–brain barrier.

**Methods:**

European Organisation for Research and Treatment of Cancer 1709/Canadian Cancer Trials Group CE.8 was a multicenter, randomized, controlled, open-label phase 3 superiority trial. Key eligibility criteria included newly diagnosed glioblastoma, age > 18 years and Karnofsky performance status > 70. Patients were randomized in a 1:1 ratio. The primary objective was to compare overall survival (OS) in patients receiving marizomib in addition to TMZ/RT→TMZ with patients receiving the only standard treatment in the whole population and in the subgroup of patients with MGMT promoter-unmethylated tumors.

**Results:**

The trial was opened at 82 institutions in Europe, Canada, and the U.S. A total of 749 patients (99.9% of the planned 750) were randomized. OS was not different between the standard and the marizomib arm (median 17 vs. 16.5 months; HR = 1.04; *P* = .64). PFS was not statistically different either (median 6.0 vs. 6.3 months; HR = 0.97; *P* = .67). In patients with *MGMT* promoter-unmethylated tumors, OS was also not different between standard therapy and marizomib (median 14.5 vs. 15.1 months, HR = 1.13; *P* = .27). More CTCAE grade 3/4 treatment-emergent adverse events were observed in the marizomib arm than in the standard arm.

**Conclusions:**

Adding marizomib to standard temozolomide-based radiochemotherapy resulted in more toxicity, but did not improve OS or PFS in patients with newly diagnosed glioblastoma.

**Key Points**

- Marizomib is a novel pan-proteasome inhibitor that crosses the blood–brain barrier.
- EORTC 1709/CCTG CE.8 (MIRAGE) is the first phase 3 trial exploring a proteasome inhibitor in patients with glioblastoma.
- The addition of marizomib to standard temozolomide-based radiochemotherapy did not prolong overall survival in patients with glioblastoma.

**Importance of the Study**

This randomized phase 3 trial addressed the question of the addition of marizomib, a novel proteasome inhibitor that crosses the blood–brain barrier, to standard temozolomide-based radiochemotherapy prolongs the survival of patients with newly diagnosed glioblastoma compared to patients receiving standard treatment alone. The study enrolled 749 patients. Despite crossing the blood–brain barrier, additional treatment with marizomib had no beneficial effect in this patient population. More research is needed to understand how proteasome inhibition may be exploited to achieve a clinical benefit for patients with glioblastoma.

Treatment options for patients with newly diagnosed glioblastoma remain limited and comprise maximal safe surgery, radiation therapy and concomitant, and maintenance treatment with the alkylating agent, temozolomide.^1,2^ In some countries, treatment with tumor-treating fields has become available as an additional treatment modality.^3^ The last decade has seen a failure of various approaches to prolong the survival of patients with newly diagnosed glioblastoma. These include targeted agents such as the anti-vascular endothelial growth factor (VEGF) antibody bevacizumab,^4,5^ the integrin antagonist cilengitide,^6^ the antibody–drug conjugate depatuxizumab mafodotin that binds EGFR^7^ as well as rindopepimut, a peptide vaccine targeting EGFRvIII.^8^ More recently, the PD-1 inhibitor nivolumab was assessed in 3 randomized trials for patients with newly diagnosed or recurrent glioblastoma and did not confer a clinical benefit in any of these studies.^9–11^ Furthermore, the combination of nivolumab with ipilimumab was not superior to temozolomide in patients with MGMT promoter-unmethylated newly diagnosed glioblastoma.^12^

Because of the disappointing results obtained with angiogenesis inhibitors and immunotherapeutic agents, other therapeutic strategies have gained increasing interest. In this regard, the proteasome has long been considered a promising target for anti-cancer therapy.^13^ Proteasome inhibitors are approved for multiple myeloma and have been explored in the glioma field for more than 2 decades. Bortezomib, the first clinically approved proteasome inhibitor against multiple myeloma, did not show clinical activity against glioblastoma, which was ascribed to the poor crossing of the blood–brain barrier.^14^ In contrast to other proteasome inhibitors, marizomib was identified as a novel inhibitor targeting all enzymatic sites of the proteasome. Preclinical studies indicate that the drug crosses the blood–brain barrier and exerts anti-glioma activity in animal models.^15,16^ In line with this, clinical activity of marizomib was observed in multiple myeloma patients with tumor manifestations in the CNS.^17^ Based on these findings, marizomib was tested in a phase 1/2 clinical trial in patients with recurrent glioblastoma, either as a single agent or in combination with bevacizumab with preliminary findings suggesting particularly good activity in patients with MGMT promoter-unmethylated tumors.^18^ In parallel, marizomib was investigated in combination with radiotherapy and temozolomide chemotherapy in a phase 1 trial in patients with newly diagnosed glioblastoma.^19^

Based on the evidence suggesting that the proteasome is a promising therapeutic target in glioblastoma as well as the preclinical and clinical datasets on marizomib, a randomized trial was designed to assess its activity in patients with newly diagnosed glioblastoma. Here, we present the results of the phase 3 European Organisation for Research and Treatment of Cancer (EORTC) 1709/Canadian Cancer Trials Group (CCTG) CE.8 (MIRAGE) trial investigating the efficacy and safety of marizomib in combination with temozolomide-based radiotherapy and maintenance temozolomide therapy vs. radiotherapy and temozolomide alone.

## Methods

### Patients

Patients were recruited at 82 sites in 12 European countries, Canada and the U.S. Key eligibility criteria included newly diagnosed, histologically confirmed glioblastoma (WHO grade 4), age of 18 years or older and a KPS of 70% or higher. No prior treatment for glioblastoma other than surgery was allowed. Patients had to be on a stable or decreasing dose of corticosteroids for at least 1 week prior to informed consent. A brain MRI after surgery obtained within 14 days of randomization was required. Patients with a tumor known to harbor an isocitrate dehydrogenase (IDH) mutation was not eligible. IDH mutation testing was recommended for patients younger than 55 years and those with tumors with atypical features per WHO 2016 classification.^20^

The trial was registered at ClinicalTrials.gov (identifier NCT03345095) and approved by the institutional review boards of all participating institutions. The study was conducted in accordance with the Declaration of Helsinki and Good Clinical Practice. All patients gave written informed consent prior to enrollment.

### Study Design and Treatment Arms

This was an open-label, phase 3 study. Randomization was performed centrally following registration using the Medidata Rave EDC system, accessible 24 h a day, 7 days a week. Patients were stratified according to the institution, age (≤55, >55 years), KPS performance status (70/80, 90/100), and extent of surgery (partial/biopsy, gross total). No blinding procedures were applied as this was an open-label study. Eligible patients were randomized 1:1 to receive radiotherapy (60 Gy in 30 fractions over 6 weeks) with concomitant temozolomide (75 mg/m^2^ once daily for 6 weeks followed by a 4-week treatment break) followed by maintenance treatment with temozolomide (150–200 mg/m^2^ once daily on days 1–5 of a 28-day cycle for up to 6 cycles) or the same treatment regimen and additional marizomib therapy. Marizomib was given as a 10 min infusion i.v., at a starting dose of 0.8 mg/m^2^ on days 1, 8, 15, 29, and 36 during radiotherapy and on days 1, 8, and 15 of each 28-day cycle during maintenance therapy with temozolomide. After completion of 6 cycles of temozolomide maintenance therapy, marizomib was given on days 1, 8, and 15 of each 28-day cycle for another 12 cycles. Marizomib was administered until progression, unacceptable toxicity, withdrawal of consent, or for a maximum of 12 additional cycles following completion of maintenance temozolomide therapy (resulting in a maximum of 18 marizomib cycles). In the event that 1 drug (temozolomide or marizomib) had to be discontinued for reasons other than disease progression, the other was continued as a single agent at the investigator discretion.

### Study Endpoints

The primary endpoint of the trial was overall survival (OS). Secondary endpoints included progression-free survival (PFS) based on Response Assessment in Neuro-Oncology (RANO) criteria, best overall response, objective response rate, complete response rate, duration of response, frequencies, and percentages of worst adverse events (AEs), or laboratory event grades, quality of life, and cognition. Key exploratory endpoints included the activity of the proteasome in the tumor tissue prior to treatment start and correlation with patients’ outcomes.

### Outcome Measures

OS was defined as the number of days from the date of randomization to the date of death due to any cause. If a patient had not died at the date of the analysis, the data were censored at the last date documented to be alive. PFS was defined as the number of days from the date of randomization to the earliest date of disease progression or to the date of death due to any cause if progression was not reported. The data were censored at the last date of imaging without documentation of progression.

### Assessments

Formalin-fixed paraffin-embedded (FFPE) tumor tissue specimens were collected at IBBL (Integrated BioBank Luxembourg). The *MGMT* promoter methylation status was centrally determined at HistoGeneX. Radiographic tumor assessments were performed by the investigators using contrast-enhanced MRI according to RANO criteria. MRI evaluation was done at baseline, 4 weeks (±7 days) after completion of radiotherapy and then every 8 weeks (±7 days) until progression. Adverse events were assessed continuously from informed consent signature up to 28 days after the end of treatment according to National Cancer Institute Common Terminology Criteria for Adverse Events (CTCAE) version 5.0. Health-related quality of life was assessed using the EORTC QLQ-C30, and EORTC QLQ-BN20 scales. Cognition was assessed using the Mini Mental State Examination (MMSE).

### Statistical Design

This study was a multicenter, randomized, controlled, open-label phase 3 superiority trial with an early stopping rule for futility. After signing the informed consent form and upon confirmation of patient eligibility, patients were randomized 1:1 to the experimental arm or the standard arm. For the study design, we assumed that the marizomib arm presents with a superior OS compared to the standard arm. The expected effect was a hazard ratio (HR) between arms equal to 0.74 (26% reduction of the hazard of death) corresponding to a median OS of 16 months in the standard arm compared to 21.6 months in the marizomib arm.^4,5^

We also assumed that at the time of final analysis, the *MGMT* promoter methylation status would be distributed according to 60% unmethylated, 30% methylated and 10% undetermined. We also hypothesized that the marizomib effect would be mainly present in the subgroup of patients with *MGMT* promoter-unmethylated tumors with an HR equal to 0.70 corresponding to a median OS of 13 months in the standard arm and 18.6 months in the marizomib arm. The effect in the *MGMT* promoter-methylated subgroup was assumed not clinically relevant (HR > 0.80) and the *MGMT*-undetermined cases were assumed to be a balanced mixture of patients with *MGMT* promoter-unmethylated and -methylated tumors with an HR equal to 0.74. For the primary OS analysis, the treatment effect was measured in the intent-to-treat (ITT) population and in the *MGMT* promoter-unmethylated subgroup (uMGMT). The estimated number of deaths in the ITT population needed to show the treatment effect on OS with 86% power and one-sided 1.5% significance was 488. This number was 320 deaths in the uMGMT cohort with 80.7% power and a one-sided 1% significance. A graphical method was used to control overall type 1 error at one-sided 2.5%, implying that if one of the analyses in a population was statistically significant, its significance was allocated to the other population.^21^

Accrual was estimated to be 150 ITT patients in the first year, and then 400 ITT patients per year thereafter. We planned to recruit 750 ITT patients in about 30 months and follow them up for a minimum of 19 months, the time necessary to observe the required number of deaths in both the ITT and uMGMT groups.

An Independent Data Monitoring Committee (IDMC) was appointed to review study safety and efficacy data. A first IDMC meeting was planned after the randomization of the first 100 ITT patients with a minimum of 3 months of follow-up for safety review only. A nonbinding futility interim analysis was planned in the ITT population when 406 patients were randomized. At that time, at least 88 deaths had to be observed. If the observed HR was larger than 1.12 then the study could be stopped for futility. There was no plan to perform an interim analysis in the uMGMT subgroup. At the first safety and tolerability IDMC meeting, the committee expressed some concerns regarding patient safety and recommended urging vigilance regarding the occurrence of encephalopathy. The committee requested a second safety analysis after 200 patients were evaluated. At the second IDMC meeting, the committee noted that marizomib had significant toxicities which were likely to impact patients’ quality of life. However, the level of toxicity was comparable to other targeted cancer drugs, and in the event that the drug proved to be efficacious, would not a priori preclude its use. It would be for future patients and doctors to decide whether the benefits justify the toxicity. At the time of the futility analysis, 616 patients were randomized, and 106 deaths were reported as of the cut-off date of 28 April 2020. Although the primary efficacy results did not cross the prespecified futility boundary, the IDMC observed that there was as yet no evidence that marizomib provided a benefit in survival and that it was extremely unlikely that a difference would emerge with additional patients or further follow-up. The committee observed that marizomib induced neurological and neuropsychiatric disorders, as well as other treatment-related adverse events, in a substantial proportion of patients. The IDMC therefore recommended that recruitment into the study be terminated. Further to the recommendations of the IDMC, patient recruitment was prematurely closed. The total number of randomized patients was 749 (750 planned). The interim analysis was considered as the final analysis of this study with key results presented at the 2021 ASCO Annual Meeting.^22^ However, all randomized patients were followed up until at least the total number of OS events initially planned for the final analysis was observed (488 deaths). That mature follow-up analysis is presented in this manuscript.

### Statistical Analysis

Frequency tables are tabulated (by treatment group) for all categorical variables by the levels of the variables as they appear on the CRF (with %). Continuous variables are presented using the median and range (minimum, maximum). For OS, the HR (including two-sided 97 % CI in the ITT and 98% CI in the uMGMT subgroup) of the marizomib arm over the standard arm are calculated by Cox’s proportional hazards model stratified by the stratification factors assessed at randomization (except institution). Kaplan–Meier survival curves (product-limit estimates) are presented by the treatment group, together with a summary of associated statistics including their two-sided 95% confidence. All efficacy analyses are performed in the ITT population (ie all patients randomized according to their allocated treatment) and for both MGMT methylation cohorts. The same calculations are performed for PFS analyses, HR was shown with a two-sided 95% CI.

As sensitivity analyses, all OS, PFS and response analyses are repeated per protocol (ie all randomized patients who were eligible, started their allocated treatment and had no major protocol deviations as defined in the medical review plan) at 5% significance.

All safety analyses are performed in the safety population (ie all randomized patients who had started any treatment arm and had received at least 1 dose of treatment. If a patient received a treatment other than the subject’s randomized treatment arm, then the patient was assigned to the treatment arm that the subject actually received during the study).

### Role of the Funding Source

All data was reviewed by EORTC staff and the first author. EORTC was the study sponsor and vouched for the integrity, accuracy, and completeness of data. All analyses were performed by the investigators and EORTC staff. Celgene/BMS provided marizomib free of charge and supported the trial through an unrestricted grant. The company had no role in data collection, data analysis, data interpretation, or writing of the manuscript. All authors had full access to the data in the study and had final responsibility for the decision to submit for publication.

## Results

**Figure 1.**
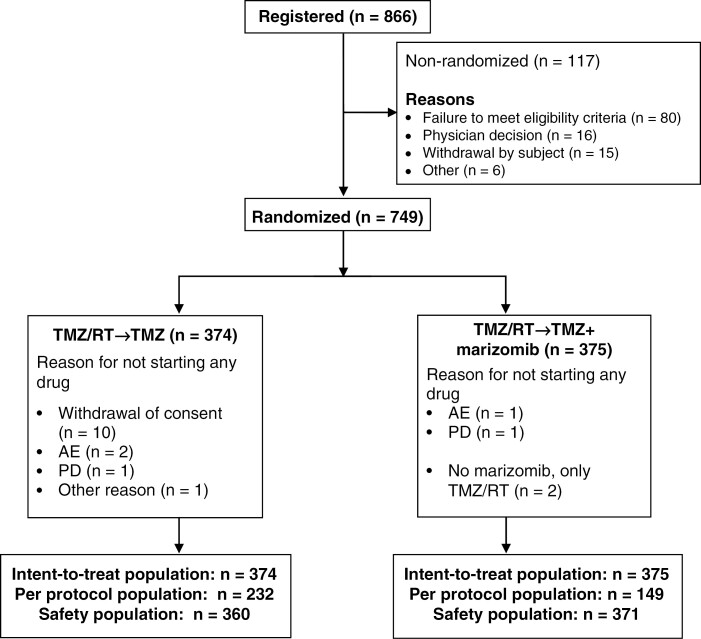
Consort chart. Abbreviations: AE, adverse event; ITT, intention-to-treat; PD, progressive disease; PP, per protocol; RT, radiotherapy; TMZ, temozolomide

From July 2018 through September 2020, 866 patients were recruited at 82 sites across 12 countries. One hundred seventeen (117) patients were screen failures (Figure 1). Of 749 patients who were randomized, 375 patients (50.1%) were assigned to receive standard treatment plus marizomib, and 374 patients (49.9%) to receive standard radiochemotherapy. In September 2020, when enrollment was close to completion (749 out of 750 patients enrolled), the investigators were informed that no additional patients were to be enrolled because of a lack of efficacy in the experimental arm and a higher percentage of AEs in the experimental arm according to a preplanned interim analysis.

Patient characteristics were balanced between the 2 treatment arms, including age, baseline KPS, and use of corticosteroids at enrolment (Table 1). There was a numerically higher proportion of males in the standard treatment arm (68.2% vs. 62.1%). In both treatment arms, approximately 50% of patients had undergone gross total resection, around 40% had partial resection in each arm and the remaining patients underwent biopsy only. One patient in the marizomib arm had a tumor that was IDH^R132H^-positive as determined by immunohistochemistry. Stratification factors were similarly distributed between treatment arms.

**Table 1.** Patient Characteristics

	TMZ/RT → TMZ (*N* = 374)	TMZ/RT → TMZ + Marizomib (*N* = 375)	Total (*N* = 749)
	*N* (%)	*N* (%)	*N* (%)
**Age (years)**			
** Median**	58.5	58.0	58.0
** Range**	21.0 – 79.0	20.0–79.0	20.0–79.0
**Sex**			
** Male**	255 (68.2)	233 (62.1)	488 (65.2)
** Female**	119 (31.8)	142 (37.9)	261 (34.8)
**Extent of resection**			
** Biopsy**	38 (10.2)	30 (8.0)	68 (9.1)
** Partial resection**	146 (39.0)	154 (41.1)	300 (40.1)
** Gross total resection**	190 (50.8)	191 (50.9)	381 (50.9)
**Corticosteroids at baseline**			
** No**	215 (57.5)	225 (60.0)	440 (58.7)
** Yes**	159 (42.5)	150 (40.0)	309 (41.3)
**KPS**			
** 90/100**	249 (66.6)	252 (67.2)	501 (66.9)
** 70/80**	125 (33.4)	123 (32.8)	248 (33.1)
***MGMT* promoter**			
** Unmethylated**	233 (59.6%)	217 (57.9%)	440 (58.7%)
** Methylated**	116 (31%)	122 (32.5%)	238 (31.8%)
** Undetermined/** **Invalid**	35 (9.4)	36 (9.6)	71 (9.5)
**IDH1** ^**R132H**^ **immunohistochemistry**			
** Negative**	287 (76.7)	287 (76.5)	574 (76.6)
** Positive**	0 (0.0)	1 (0.3)	1 (0.1)
** Undetermined**	1 (0.3)	3 (0.8)	4 (0.5)
** Not done**	86 (23.0)	84 (22.4)	170 (22.7)
**MMSE**			
** ≥27**	277 (74.1)	281 (74.9)	558 (74.5)
** <27**	68 (18.2)	70 (18.7)	138 (18.4)
** Missing**	29 (7.8)	24 (6.4)	53 (7.1)

Abbreviations: IDH, isocitrate dehydrogenase; KPS, Karnofsky Performance Status; MGMT, O^6^-methylguanine DNA-methyltransferase; MMSE, mini-mental state examination; RT, radiotherapy; TMZ, temozolomide.

Fourteen patients in the standard arm (10 withdrew consent, 2 had AEs, 1 had early progressive disease, and 1 other reason) and 2 patients in the marizomib arm (1 AE and 1 progressive disease) did not start any drug. Two patients in the marizomib arm received TMZ/RT→TMZ only (Figure 1).

Ninety-six percent (96%) of patients received at least 90% of the RT dose (95% in the marizomib arm and 97% in the standard arm). The median relative dose intensity of concomitant TMZ delivery was 99% in the marizomib group and 99.6% in the standard therapy group. The median relative dose intensity of concomitant marizomib delivery was 99%. Maintenance TMZ was started in 83% of patients in the marizomib arm and 84% of patients in the standard arm. However, maintenance marizomib therapy was started in only 72% of patients in the experimental arm. In patients who started maintenance therapy with TMZ, the median number of TMZ cycles received by patients in the marizomib arm was 6 (range 1–14). The median number of maintenances TMZ cycles received by patients in the standard arm was 5 (range 1–12). Patients in the marizomib group had a median of 4 maintenance marizomib cycles (range 1–18).

At the time of this long-term follow-up analysis, TMZ administration had been discontinued in all patients in both arms. Marizomib administration was discontinued in all patients in the experimental arm. The main reasons for temozolomide discontinuation were progressive disease (49% in the standard arm and 47% in the marizomib arm), completion of maintenance therapy (33% in the standard arm and 32% in the marizomib arm) and adverse events (10% in the standard arm and 10% in the marizomib arm). The main reasons for marizomib discontinuation were progressive disease (42%), adverse events (32%) and patient withdrawal (16%).

### Efficacy

At the time of this long-term follow-up analysis, 538 patients out of 749 randomized had died and the median follow-up time for OS was 29.1 months (95% CI, 26.3–30.5 months) in the marizomib arm and 27.5 months (95% CI, 26.1–28.6) in the standard arm (log-rank test: *P* = 0.42).

**Figure 2.**
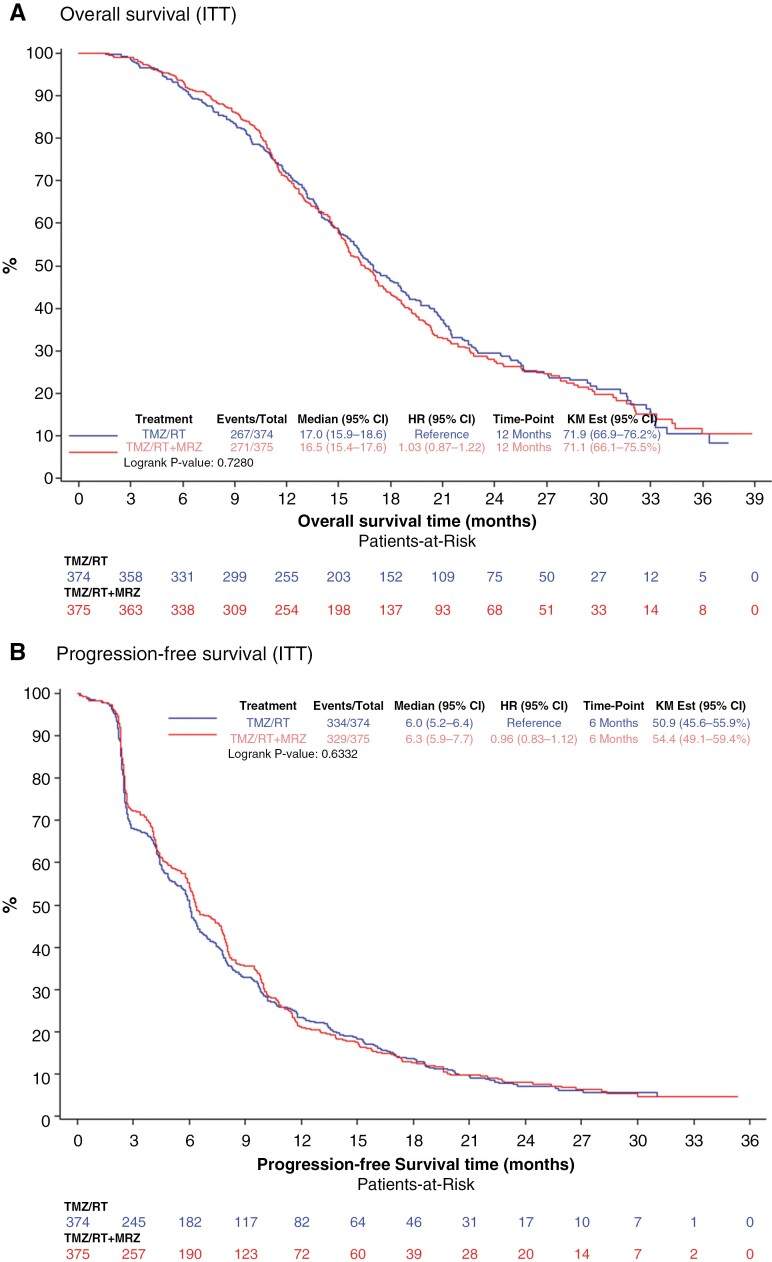
Overall survival and progression-free survival in the ITT population. A. Kaplan–Meier curves, number of events and median overall survival are shown for patients treated with standard therapy alone (TMZ/RT) or standard therapy plus marizomib (TMZ/RT + marizomib). Hazard ratios and confidence intervals were estimated using a Cox proportional hazards model. B. Kaplan–Meier curves, number of events and median progression-free survival are shown for patients treated with standard therapy alone (TMZ/RT) or standard therapy plus marizomib (TMZ/RT + marizomib). Hazard ratios and confidence intervals were estimated using a Cox proportional hazards model.

The overall survival in the ITT population was not statistically significant between the standard arm and the marizomib arm (median 17 months (95% CI, 15.9–18.6) vs. 16.5 months (95% CI, 15.4–17.6); stratified hazard ratio HR = 1.04; 95% CI, 0.86–1.27, stratified log-rank: p = 0.64). The overall survival at 12 months was 71.1% (95% CI, 66.1–75.5) in the marizomib arm and 71.9% (95% CI, 66.9–76.2) in the standard arm (Figure 2A).

Progression-free survival in the ITT population was not statistically different (median 6.3 months in the marizomib arm (95% CI, 5.9–7.7) and 6.0 months in the standard arm (95% CI, 5.2–6.4); stratified HR = 0.97; 95% CI, 0.82–1.13, stratified log-rank: *P* = .67) (Figure 2B).

**Figure 3.**
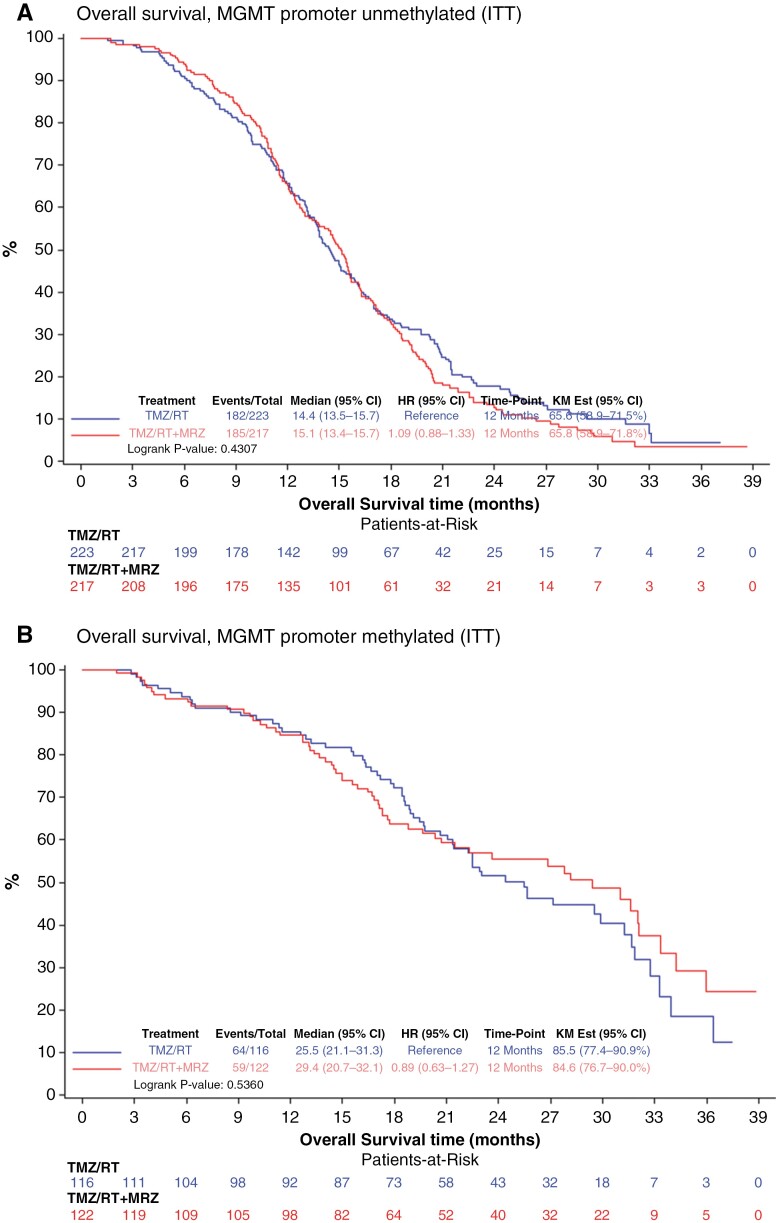
Overall survival stratified for *MGMT* promoter methylation status. Kaplan–Meier curves, number of events and median overall survival are shown for patients treated with standard therapy alone (TMZ/RT) or standard therapy plus marizomib (TMZ/RT + marizomib). Hazard ratios and confidence intervals were estimated using a Cox proportional hazards model. A. Patients with *MGMT* promoter-unmethylated tumors. B. Patients with *MGMT* promoter-methylated tumors.

**Figure 4.**
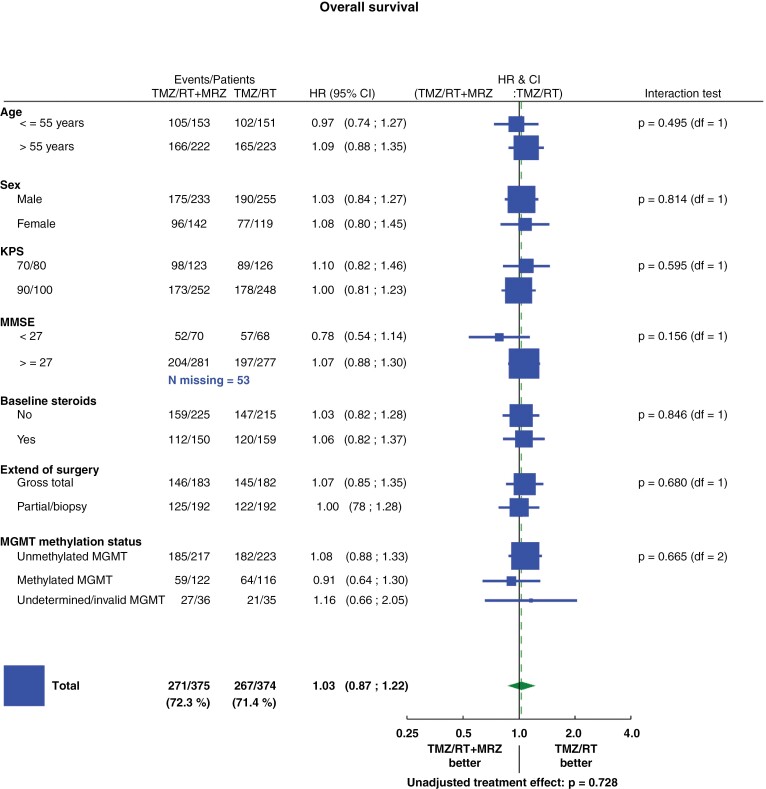
Overall survival in prespecified patient subgroups is defined by baseline clinical characteristics. Forest plot showing hazard ratios for death in the analysis of treatment effect in prespecified patient subgroups according to baseline characteristics.

Preliminary data from the recurrent glioblastoma trial suggested that treatment with marizomib might be particularly beneficial in the subgroup of patients with *MGMT* promoter-unmethylated tumors.^18^ OS in patients with *MGMT* promoter-unmethylated tumors in the ITT population (ITT/uMGMT) was not statistically different (median 15.1 months (95% CI, 13.4–15.7) in the marizomib arm and 14.5 months (95% CI, 13.5–15.7) in the standard arm; stratified HR = 1.13; 95% CI, 0.88–1.44, stratified log-rank: *P* = .27) (Figure 3A). Among patients with a tumor harboring *MGMT* promoter methylation in the ITT population (ITT/mMGMT), OS was not statistically different (median OS 29.4 months (95% CI, 20.7–32.1) in the marizomib arm and 25.5 months (95% CI, 21.1–31.3) in the standard arm; stratified HR = 0.86; 95% CI, 0.60–1.24, stratified log-rank: *P* = 0.41) (Figure 3B). In the ITT/uMGMT population, PFS was not statistically different (median PFS 5.9 months (95% CI, 4.6–6.4) in the marizomib arm and 5.1 months (95% CI, 4.4–6.0) in the standard arm; stratified HR = 0.96; 95% CI, 0.78–1.16, stratified log-rank: *P* = .64) (Supplementary Figure 1A). In the ITT/mMGMT population, PFS was not statistically different (median PFS 10.4 months (95% CI, 8.2–11.7) in the marizomib arm and 10.0 months (95% CI, 8.0–13.1) in the standard arm; stratified HR = 0.96; 95% CI, 0.71–1.29, stratified log-rank: *P* = .79) (Supplementary Figure 1B). There was no statistically significant difference in the treatment effect on OS by the prespecified subgroup (Figure 4).

In the standard arm, 232 patients (62%) and in the marizomib arm 149 patients (40%) were included in the per protocol population. In the marizomib arm, the main reason for exclusion was major protocol violation (*n* = 193, 52%), mainly because of the absence of ECG evaluation, which was implemented in protocol version 5.1 according to an FDA recommendation but not done in all patients. There was no such recommendation for patients in the standard arm. All efficacy results were similar in the per protocol population compared to the ITT population indicating no advantage of the marizomib arm over the standard arm.

Investigators were free to decide on subsequent therapy at the end of the study therapy. In the marizomib arm, 61% of patients had second-line treatment, including surgery (16%), re-irradiation (13%) or systemic treatment (57%), including bevacizumab (28%), TMZ (21%), and lomustine (25%). In the standard arm, 64% of patients received further treatment including surgery (18%), re-irradiation (14%), and 60% had systemic therapy including bevacizumab (27%), TMZ (18%), and lomustine (29%).

### Safety and Toxicity

The 2 patients in the marizomib arm who received TMZ/RT→TMZ only (Figure 1), were considered as part of the safety population ie these patients were reallocated to the standard arm for all safety analyses. In the marizomib arm, patients were more frequently affected by grade 3 or 4 Treatment-Emergent Adverse Events (TEAE). In particular, patients in the marizomib arm had more (>5% difference) grade 3 or 4 AE than patients in the standard arm for the following AE: any grade 3/4 AE: 67% in the marizomib arm (55% related, 39% serious, 25% related and serious, 48% leading to dose reduction, 55% leading to dose interruption, and 36% leading to dose withdrawal) and 48% of patients in the standard arm (27% related, 27% serious, 12% related and serious, 7% leading to dose reduction, 29% leading to dose interruption, and 12% leading to dose withdrawal). In the marizomib arm, 8 patients died from AEs (2 from soft tissue necrosis, 1 each: intestinal perforation, seizure, cerebral hemorrhage, leukoencephalopathy, bacterial meningitis, and head injury). In the standard arm, 1 patient died of febrile neutropenia. The hematological and biochemistry toxicity profiles were not different between the 2 treatment arms. Grade 3/4 AEs affecting the nervous system were observed in 33% of patients in the marizomib arm and 20% of patients in the standard arm. One suicide attempt and 1 “assisted” suicide attempt were reported in the marizomib arm. Grade 3/4 psychiatric disorders were reported in 14% of patients in the marizomib arm and 3% of patients receiving standard therapy alone (Table 2). All neurological and psychiatric AEs could be managed by dose delays and dose reductions.

**Table 2.** Adverse Events

	TMZ/RT → TMZ (Safety Population, *n* = 362)					TMZ/RT → TMZ + marizomib (Safety Population, *n* = 371)				
System Organ Class + Clinical Description	Grade 3 *N* (%)	Grade 4 *N* (%)	Grade 5 *N* (%)	Grade 3/4 *N* (%)	Grade ≥1 *N* (%)	Grade 3 *N* (%)	Grade 4 *N* (%)	Grade 5 *N* (%)	Grade 3/4 *N* (%)	Grade ≥1 *N* (%)
Patients’ worst grade	137 (37.8)	37 (10.2)	4 (1.1)	174 (48.1)	358 (98.9)	210 (56.6)	40 (10.8)	8 (2.2)	250 (67.4)	371 (100.0)
Blood and lymphatic system disorders	24 (6.6)	10 (2.8)	1 (0.3)	34 (9.4)	78 (21.5)	24 (6.5)	13 (3.5)		37 (10)	89 (24)
Thrombocytopenia	11 (3)	7 (1.9)		18 (5)	43 (11.9)	10 (2.7)	8 (2.2)		18 (4.9)	44 (11.9)
Gastrointestinal disorders	12 (3.3)			12 (3.3)	244 (67.4)	34 (9.2)	1 (0.3)		35 (9.4)	322 (86.8)
Constipation	2 (0.6)			2 (0.6)	123 (34)	1 (0.3)			1 (0.3)	157 (42.3)
Diarrhea	3 (0.8)			3 (0.8)	39 (10.8)	3 (0.8)			3 (0.8)	62 (16.7)
Nausea	3 (0.8)			3 (0.8)	159 (43.9)	15 (4)			15 (4)	260 (70.1)
vomiting	4 (1.1)			4 (1.1)	70 (19.3)	20 (5.4)			20 (5.4)	206 (55.5)
General Disorders	28 (7.7)	3 (0.8)	1 (0.3)	31 (8.6)	272 (75.1)	71 (19.1)	2 (0.5)		73 (19.7)	318 (85.7)
Asthenia	1 (0.3)			1 (0.3)	38 (10.5)	5 (1.3)			5 (1.3)	47 (12.7)
Fatigue	9 (2.5)			9 (2.5)	218 (60.2)	34 (9.2)			34 (9.2)	243 (65.5)
Gait disturbance	3 (0.8)			3 (0.8)	25 (6.9)	17 (4.6)			17 (4.6)	100 (27)
Pyrexia					18 (5)					42 (11.3)
Injury and procedural complications	9 (2.5)	1 (0.3)		10 (2.8)	77 (21.3)	11 (3)		1 (0.3)	11 (3)	106 (28.6)
Fall	5 (1.4)			5 (1.4)	21 (5.8)	6 (1.6)			6 (1.6)	54 (14.6)
Radiation skin injury					41 (11.3)					21 (5.7)
Investigations	26 (7.2)	20 (5.5)		46 (12.7)	145 (40.1)	39 (10.5)	7 (1.9)		46 (12.4)	168 (45.3)
Platelet count decreased	8 (2.2)	16 (4.4)		24 (6.6)	55 (15.2)	8 (2.2)	4 (1.1)		12 (3.2)	41 (11.1)
Weight decreased	2 (0.6)			2 (0.6)	35 (9.7)					65 (17.5)
Metabolism and nutrition disorders	8 (2.2)	1 (0.3)		9 (2.5)	90 (24.9)	13 (3.5)	2 (0.5)		15 (4)	126 (34)
Decreased Appetite					63 (17.4)					81 (21.8)
Musculoskeletal and connective tissue disorders	4 (1.1)			4 (1.1)	88 (24.3)	12 (3.2)	1 (0.3)	1 (0.3)	13 (3.5)	139 (37.5)
Muscular weakness	2 (0.6)			2 (0.6)	29 (8)	9 (2.4)			9 (2.4)	50 (13.5)
Nervous system disorders	68 (18.8)	5 (1.4)	1 (0.3)	73 (20.2)	265 (73.2)	118 (31.8)	6 (1.6)	4 (1.1)	124 (33.4)	332 (89.5)
Aphasia	6 (1.7)			6 (1.7)	33 (9.1)	10 (2.7)			10 (2.7)	63 (17)
Ataxia	1 (0.3)			1 (0.3)	3 (0.8)	19 (5.1)			19 (5.1)	92 (24.8)
Balance disorder	1 (0.3)			1 (0.3)	11 (3)	5 (1.3)			5 (1.3)	52 (14)
Dizziness	1 (0.3)			1 (0.3)	39 (10.8)	9 (2.4)			9 (2.4)	105 (28.3)
Dysarthria					14 (3.9)	3 (0.8)			3 (0.8)	66 (17.8)
Headache	6 (1.7)			6 (1.7)	130 (35.9)	13 (3.5)			13 (3.5)	206 (55.5)
Seizure	18 (5)		1 (0.3)	18 (5)	55 (15.2)	18 (4.9)	2 (0.5)	1 (0.3)	20 (5.4)	57 (15.4)
Psychiatric disorders	9 (2.5)	1 (0.3)		10 (2.8)	124 (34.3)	43 (11.6)	10 (2.7)		53 (14.3)	277 (74.7)
Anxiety	3 (0.8)			3 (0.8)	25 (6.9)	2 (0.5)			2 (0.5)	40 (10.8)
Confusional state	4 (1.1)			4 (1.1)	32 (8.8)	10 (2.7)	2 (0.5)		12 (3.2)	82 (22.1)
Hallucination	1 (0.3)			1 (0.3)	3 (0.8)	20 (5.4)	5 (1.3)		25 (6.7)	152 (41)
Insomnia					46 (12.7)	6 (1.6)			6 (1.6)	106 (28.6)
Skin and subcutaneous tissue disorders	2 (0.6)			2 (0.6)	157 (43.4)	4 (1.1)	1 (0.3)		5 (1.3)	155 (41.8)
Alopecia					112 (30.9)					91 (24.5)

Abbreviations: RT, radiotherapy; TMZ, temozolomide.

## Discussion

Developing novel treatment options for patients with glioblastoma remains an ongoing challenge in clinical neuro-oncology. The high expectations on progress with immunotherapeutic strategies have not been met over the last years as several phase 3 trials failed to show a clinical benefit.^8–11^ The emergence of new drugs that cross the blood–brain barrier is of particular interest in the glioma field as limited drug concentrations at the tumor site in the CNS have either precluded clinical development or yielded disappointing results in the past. Marizomib fulfilled the criteria of a drug that: (i) penetrates the brain, (ii) targets the proteasome, which is a central regulator in tumor cells, and (iii) was successfully tested preclinically and in early clinical trials.^15,18,19,23,24^

Despite these promising baseline considerations, EORTC 1709 provided no evidence that the addition of marizomib translated into a statistically or clinically significant benefit in survival over standard treatment alone in patients with newly diagnosed glioblastoma (Figure 2). Furthermore, exposure to marizomib was associated with more grade 3/4 adverse events, notably neurological and psychiatric disorders, providing further clinical evidence of blood–brain barrier penetration. The administration of subsequent therapies was similar between the 2 arms and no other confounding factors explained the lack of efficacy.

Preliminary data from previous trials suggested that marizomib might be particularly active relative to historical controls in patients with MGMT promoter-unmethylated tumors. Therefore, OS in this subgroup was defined as a coprimary endpoint. In the ITT population, there were 58.7% of patients with MGMT-promoter-unmethylated tumors, in 31.8% of cases, the MGMT promoter was methylated and in 9.5% undetermined. Per the study design, it was assumed that MGMT would be distributed 60% unmethylated, 30% methylated, and 10% undetermined. Therefore, the overall MGMT assessment is in accordance with the study design assumptions. Although MGMT was not a stratification factor at randomization, the MGMT promoter status distribution was similar between the 2 treatment arms. The population of patients with MGMT promoter-unmethylated tumors has a high unmet medical need as standard alkylating chemotherapy is typically inactive. Over the last years, several efforts have made to identify a drug that is superior to TMZ in this subgroup of patients. However, the combination of radiotherapy with a PD-1 inhibitor did not prolong survival.^9^ Similarly, the mTOR inhibitor temsirolimus did not improve survival compared to standard temozolomide-based radiochemotherapy.^25^ In the current study, the addition of marizomib did not confer a survival advantage compared to standard therapy alone in patients with MGMT promoter-unmethylated tumors either (Figure 3). In addition, there was also no beneficial effect in the cohort of patients with MGMT promoter-methylated tumors.

At the time when the study was enrolling, the 2016 version of the WHO classification of CNS tumors was still in place.^20^ Because of the foreseeable adaptions in the upcoming WHO classification and to reduce the number of patients with IDH-mutant tumors to a minimum, all patients with a tumor known to harbor an IDH mutation were not eligible for study participation. Since IDH testing, particularly sequencing, was not mandatory, it cannot be excluded that single patients with IDH-mutant gliomas were enrolled. However, it seems very unlikely that this had any significant impact on the outcome of the trial.

Administration of marizomib was associated with a higher rate of neurological and psychiatric adverse events (Table 2). As the drug crosses the blood–brain barrier, this finding was anticipated. No new safety signals were observed in this study compared to those noted in previous phase 1/2 studies in either newly diagnosed or recurrent glioblastoma.^18,26^

The reasons for the lack of activity of marizomib in the current trial remain to be determined. Crossing of the blood–brain barrier was shown in nonhuman primates.^15^ Furthermore, the occurrence of CNS adverse events following marizomib administration suggests penetration of the drug to the brain. However, it is possible that the concentrations reached at the tumor site were insufficient to interfere with the enzymatic activities of the proteasome. Resistance to marizomib, eg based on insufficient induction of cell death, that was not or only partially captured in the preclinical characterization may represent an alternative explanation.^23^ For future trials, a more thorough evaluation of new drugs in smaller trials, including phase 0 concepts in order to demonstrate sufficient drug delivery to the tumor site^27^ or an assessment in studies with a more innovative trial design such as the ongoing AGILE study (NCT03970447) should be considered.

In conclusion, adding marizomib to radiotherapy and temozolomide chemotherapy did not result in a survival benefit and was associated with additional toxicity. Within an ongoing translational research program, we aim to understanding the underlying mechanism for the failure of marizomib to confer a clinical benefit despite its strong anti-proteasome activity and ability to cross the blood–brain barrier. The availability of biomarkers that allow for the identification and selection of patients whose tumors might be amenable to proteasome inhibitor treatment in combination with other therapeutic strategies may allow for further clinical development of proteasome inhibition in clinical neuro-oncology.

## Supplementary material

Supplementary material is available online at *Neuro-Oncology* (https://academic.oup.com/neuro-oncology).

noae053_suppl_Supplementary_Figures_1
