# Supplementary material for: Marizomib for patients with newly diagnosed glioblastoma: A randomized phase 3 trial
Source: Neuro Oncol. 2024 Mar 19;26(9):1670–82. doi: 10.1093/neuonc/noae053 (PMC11376448; doi:10.1093/neuonc/noae053)

Suppl. Figure 1

A Progression-free survival, MGMT promoter unmethylated (ITT)

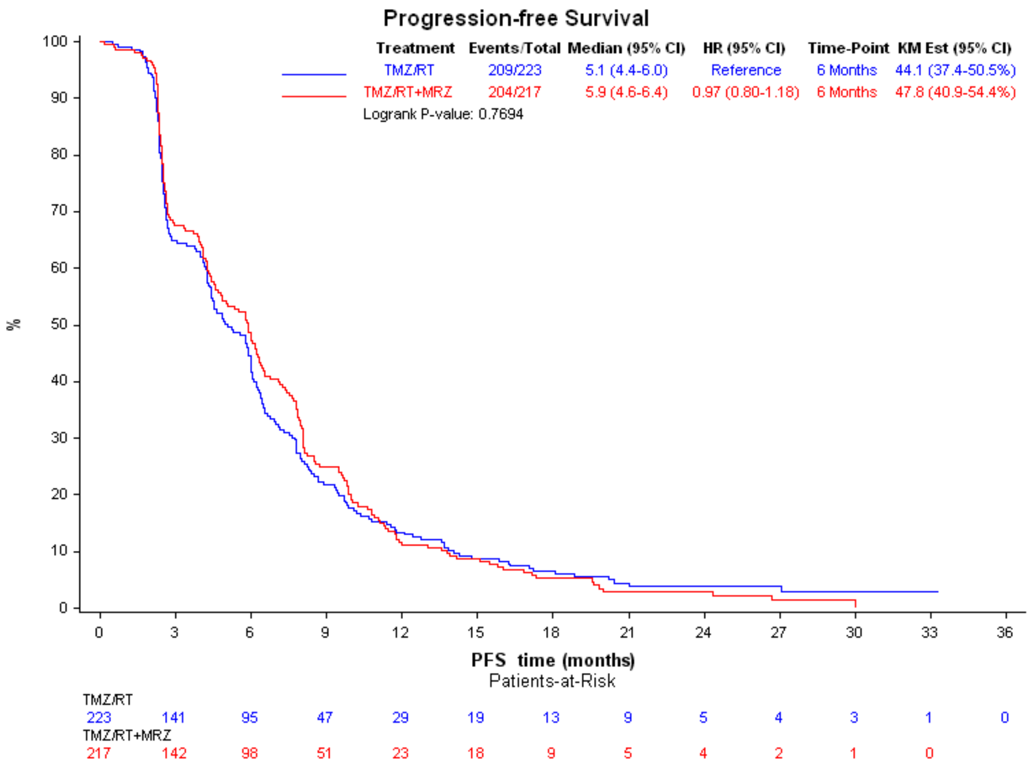

B Progression-free survival, MGMT promoter methylated (ITT)

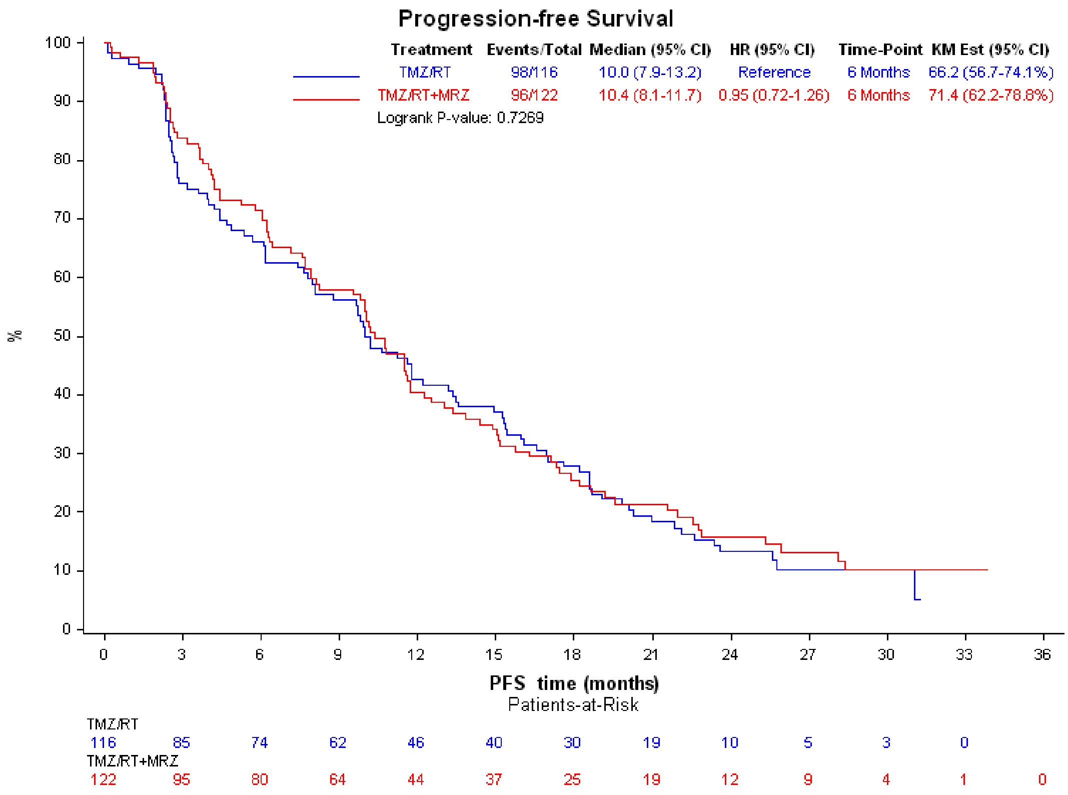

Supplement: noae053_suppl_Supplementary_Figures_1 [file noae053_suppl_supplementary_figures_1.pdf]
